# Human T-Cell Lymphotropic Virus Type 1 Subtype C Molecular Variants among Indigenous Australians: New Insights into the Molecular Epidemiology of HTLV-1 in Australo-Melanesia

**DOI:** 10.1371/journal.pntd.0002418

**Published:** 2013-09-26

**Authors:** Olivier Cassar, Lloyd Einsiedel, Philippe V. Afonso, Antoine Gessain

**Affiliations:** 1 Institut Pasteur, Unité d’Epidémiologie et Physiopathologie des Virus Oncogènes, Département de Virologie, Paris, France; 2 CNRS, UMR 3569, Paris, France; 3 Flinders University/Northern Territory Rural Clinical School, Alice Springs Hospital, Alice Springs, Northern Territory, Australia; Tulane School of Public Health and Tropical Medicine, United States of America

## Abstract

**Background:**

HTLV-1 infection is endemic among people of Melanesian descent in Papua New Guinea, the Solomon Islands and Vanuatu. Molecular studies reveal that these Melanesian strains belong to the highly divergent HTLV-1c subtype. In Australia, HTLV-1 is also endemic among the Indigenous people of central Australia; however, the molecular epidemiology of HTLV-1 infection in this population remains poorly documented.

**Findings:**

Studying a series of 23 HTLV-1 strains from Indigenous residents of central Australia, we analyzed coding (*gag, pol, env, tax*) and non-coding (LTR) genomic proviral regions. Four complete HTLV-1 proviral sequences were also characterized. Phylogenetic analyses implemented with both Neighbor-Joining and Maximum Likelihood methods revealed that all proviral strains belong to the HTLV-1c subtype with a high genetic diversity, which varied with the geographic origin of the infected individuals. Two distinct Australians clades were found, the first including strains derived from most patients whose origins are in the North, and the second comprising a majority of those from the South of central Australia. Time divergence estimation suggests that the speciation of these two Australian clades probably occurred 9,120 years ago (38,000–4,500).

**Conclusions:**

The HTLV-1c subtype is endemic to central Australia where the Indigenous population is infected with diverse subtype c variants. At least two Australian clades exist, which cluster according to the geographic origin of the human hosts. These molecular variants are probably of very ancient origin. Further studies could provide new insights into the evolution and modes of dissemination of these retrovirus variants and the associated ancient migration events through which early human settlement of Australia and Melanesia was achieved.

## Author Summary

The Human T-lymphotropic virus type 1 (HTLV-1) infects at least 5–10 million persons worldwide. In Oceania, previous studies have shown that HTLV-1 is present in a few ancient populations from remote areas of Papua New Guinea, the Solomon Islands, the Vanuatu archipelago and central Australia. The latter comprise one of the most socio-economically disadvantaged groups within any developed country. Characterization of the few available HTLV-1 viruses from Oceania indicates that these belong to a specific HTLV-1 genotype, the Australo-Melanesian c-subtype. In this study, we provide details for 23 HTLV-1 viruses derived from the Indigenous population of central Australia, a vast remote area of 1,000,000 km^2^. We reveal considerable genetic diversity of HTLV-1c subtype viruses and the existence of two HTLV-1c clades within which a high degree of genetic diversity was also apparent. These newly described HTLV-1c clades clustered according to the geographic origin of their human hosts. Indigenous Australians from the North of central Australia harbor HTLV-1c subtype viruses that are distinct from those of individuals from regions to the South. These data suggest that HTLV-1 was probably introduced to Australia during ancient migration events and was then confined to isolated Indigenous communities in central Australia.

## Introduction

The Human T-lymphotropic virus type 1 (HTLV-1) is the first described human oncoretrovirus [1]. HTLV-1 infection is associated with a universally fatal malignancy, adult T-cell leukemia/lymphoma (ATLL), and with inflammatory disorders, the prototype of which is HTLV-1 associated myelopathy/tropical spastic paraparesis (HAM/TSP) [2]. HTLV-1 infects at least 5 to 10 million people worldwide [3]. It is widely distributed, with substantial clusters of high endemicity in certain geographic areas and ethnic groups in Southwestern Japan, sub-Saharan Africa, South America, the Caribbean basin and smaller endemic foci in Iran and Australo-Melanesia [3]. Seven main molecular HTLV-1 subtypes are currently recognized, predominantly from nucleotide sequence analysis of the LTR region. These are the Cosmopolitan subtype (a) that has spread worldwide, five African subtypes (b, d-g) and an Australo/Melanesian subtype (c), which is found only in Oceania [4], [5], [6], [7], [8]. The limited horizontal transmission of HTLV-1 and its clustering in certain ethnic/geographic foci have encouraged the use of its very slow *in vivo* genetic drift as a mean of studying the origin and modes of dissemination of this retrovirus as well as the movements of ancient infected populations [4], [9], [10].

Characterization of HTLV-1c variants was initially performed by Yanagihara *et al*. among a small group of hunter-horticulturalists, the Hagahai, who live in the fringe highlands of Papua New Guinea (PNG) [11], [12], [13] and among people of Melanesian descent in the Solomon Islands [13], [14]. Subsequently, efforts have been made to characterize new HTLV-1c subtype isolates in the neighboring territories of Australia [15], [16] and the Vanuatu archipelago [9], [17]. Nevertheless, our understanding of the molecular virology of the HTLV-1c subtype remains largely based on partial genome sequences of the gp21 *env* gene and LTR regions [9], [10], [13]. Indeed, only a single complete HTLV-1c subtype nucleotide sequence has been published to date, the original MEL5 human isolate from the Solomon Islands [18]. Previous studies indicate that HTLV-1 is also endemic to central Australia where high HTLV-1 seropositivity rates have been documented among Indigenous adults admitted to the sole regional hospital [19], [20]. Indeed, cases of ATL, HAM/TSP and infective dermatitis have now been described and an association between HTLV-1 infection and bronchiectasis has also been reported among Indigenous Australians [15], [21], [22], [23], [24], [25]. Interestingly, many clinical cases arise from the same central Australian region suggesting that environmental and/or viral factors may contribute to the etiology of HTLV-1 related diseases in this population. Unfortunately, only partial HTLV-1 nucleotide sequences are available for a single HTLV-1 strain from an Indigenous Australian [15], precluding any understanding of genetic variability in this population. Establishing a large HTLV-1 sequence database is thus essential for any study of the epidemiology and pathogenicity of HTLV-1c subtype.

The aim of the present study was therefore to describe the HTLV-1 genotypes infecting Indigenous central Australian residents and to correlate the results of the HTLV-1 nucleotide sequence variability with the geographic origin of the individuals living within this vast region of approximately 1,000,000 km^2^.

## Methods

### Studied populations and data collection

**Figure 1:**
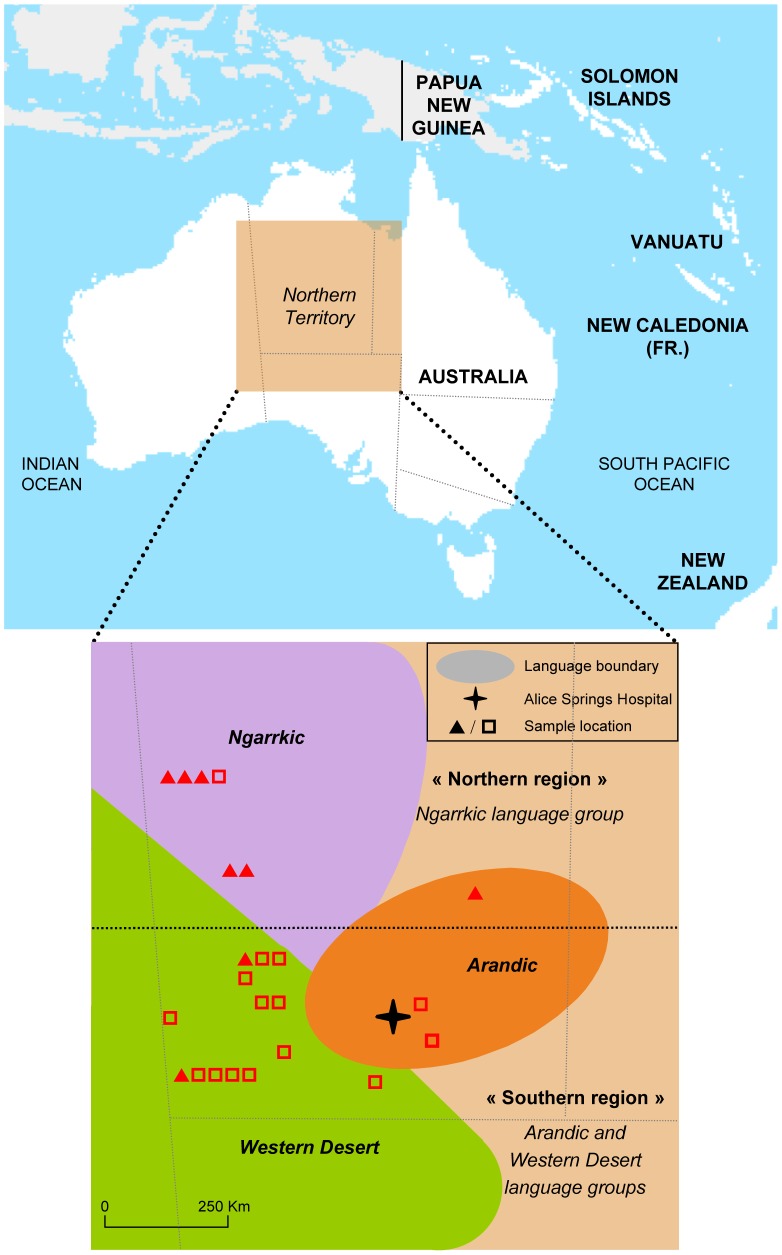
Map of central Australia. Indicated are the distribution of the three main Aboriginal language groups (Ngarrkic, Western Desert and Arandic) and the place of origin of the samples used in this study based on place of childhood residence and language (Northern, ?; Southern, ?).

Our work was performed using HTLV-1 isolates obtained from a large series of patients who were initially enrolled to HTLV-1 pathogenesis studies between October 2007 and August 2010 [22]. Plasma and peripheral blood buffy coat (PBBC) samples were obtained from 23 HTLV-1 infected patients who presented to Alice Springs Hospital, predominantly with bronchiectasis. Presumed place of origin was determined from language group and/or place of residence in infancy (figure 1). Numerous Indigenous languages are spoken in this region; however, for the purpose of the present study, these were divided into two groups according to the predominant geographic areas in which they are spoken; i) Northern (comprising the Ngarrkic language groups) and ii) Southern (comprising both Arandic and Western Desert language groups) (table 1). Also included in the present study were PBBC samples from four Natives of the Vanuatu archipelago. These were collected by us during work in Vanuatu between 2003 and 2005, as has been described previously [9], [17].

**Table 1:** Epidemiological and molecular features of HTLV-1 infected Indigenous Australians and Natives from Vanuatu.

Strain ID	Age, years	Sex	Geographical localization	Language group	HTLV-1 proviral sequenced regions	GenBank accession number
Aus-WC	27	Female	North	Ngarrkic	LTR, *gag, tax*	KC786914, KC786935, KC786956
Aus-RI	50	Male	North	Ngarrkic	LTR, *gag, tax*	KC786910, KC786931, KC786952
Aus-SQ	46	Female	North	Ngarrkic	LTR, *gag, tax*	KC786901, KC786922, KC786943
Aus-RA	46	Male	North	Ngarrkic	LTR, *gag, tax*	KC786903, KC786924, KC786945
Aus-NR	59	Female	North	Ngarrkic	Complete genome	JX891479
Aus-DF	60	Female	North	Ngarrkic	Complete genome	KF242505
Aus-WS	43	Male	North	Ngarrkic	LTR, *gag, tax*	KC786906, KC786927, KC786948
Aus-RM	52	Female	South	Arandic	LTR, *gag, tax*	KC786911, KC786932, KC786953
Aus-HJ	55	Male	South	Arandic	LTR, *gag, tax*	KC786908, KC786929, KC786950
Aus-AN	56	Male	South	Arandic	LTR, *gag, tax*	KC786905, KC786926, KC786947
Aus-GN	51	Male	South	Arandic	LTR, *gag, tax, env*	KC786899, KC786920, KC786941, KF242507
Aus-CS	29	Female	South	Arandic	Complete genome	KF242506
Aus-JM	55	Female	South	Arandic	LTR, *gag, tax*	KC786904, KC786925, KC786946
Aus-NA	47	Male	South	Arandic	LTR, *gag, tax*	KC786900, KC786921, KC786942
Aus-LT	16	Male	South	Arandic	LTR, *gag, tax*	KC786916, KC786937, KC786958
Aus-PS	31	Male	South	Western Desert	LTR, *gag, tax*	KC786918, KC786939, KC786960
Aus-GG	39	Male	South	Western Desert	LTR, *gag, tax*	KC786917, KC786938, KC786959
Aus-MI	70	Female	South	Western Desert	LTR, *gag, tax*	KC786902, KC786923, KC786944
Aus-DM	52	Female	South	Western Desert	LTR, *gag, tax*	KC786907, KC786928, KC786949
Aus-BC	30	Male	South	Western Desert	LTR, *gag, tax*	KC786919, KC786940, KC786961
Aus-CR	19	Male	South	Western Desert	LTR, *gag, tax*	KC786915, KC786936, KC786957
Aus-PC	46	Female	South	Western Desert	LTR, *gag, tax*	KC786909, KC786930, KC786951
Aus-GM	67	Male	South	Western Desert	Complete genome	JX891478
ESH18	61	Male	Vanuatu	North Central Vanuatu	LTR, *gag, tax*	KC786962, KC786966, KC786970
ESW44	40	Female	Vanuatu	North Central Vanuatu	LTR, *gag, tax*	KC786963, KC786967, KC786971
EM5	76	Female	Vanuatu	Polynesian	LTR, *gag, tax*	KC786964, KC786968, KC786972
PE376	61	Female	Vanuatu	North Central Vanuatu	LTR, *gag, tax*	KC786965, KC786969, KC786973

### Ethics statement

Written informed consent was given by all patients for their blood to be used for the pathogenicity studies, which included the molecular characterization of the HTLV-1 viral strains. The Central Australian Human Research Ethics Committee (CAHREC) approved this study (CAHREC Ref: 2011.11.01).

### HTLV-1 serologic analyses and molecular screening

The plasma and PBBC samples were transferred to Institut Pasteur, Paris, and stored at −80°C until HTLV-1 analysis. Plasma HTLV-1 antibodies were tested by a particle agglutination (PA) technique (Serodia HTLV-1, Fujirebio, Tokyo, Japan) and by an indirect immunofluorescence assay (IFA) using the HTLV-1-transformed human T cell lines MT2. All samples were also tested by Western blot assay (WB) (HTLV Blot 2.4, MP Biomedicals Asia Pacific Pte. Ltd., Singapore).

**Figure 2:**
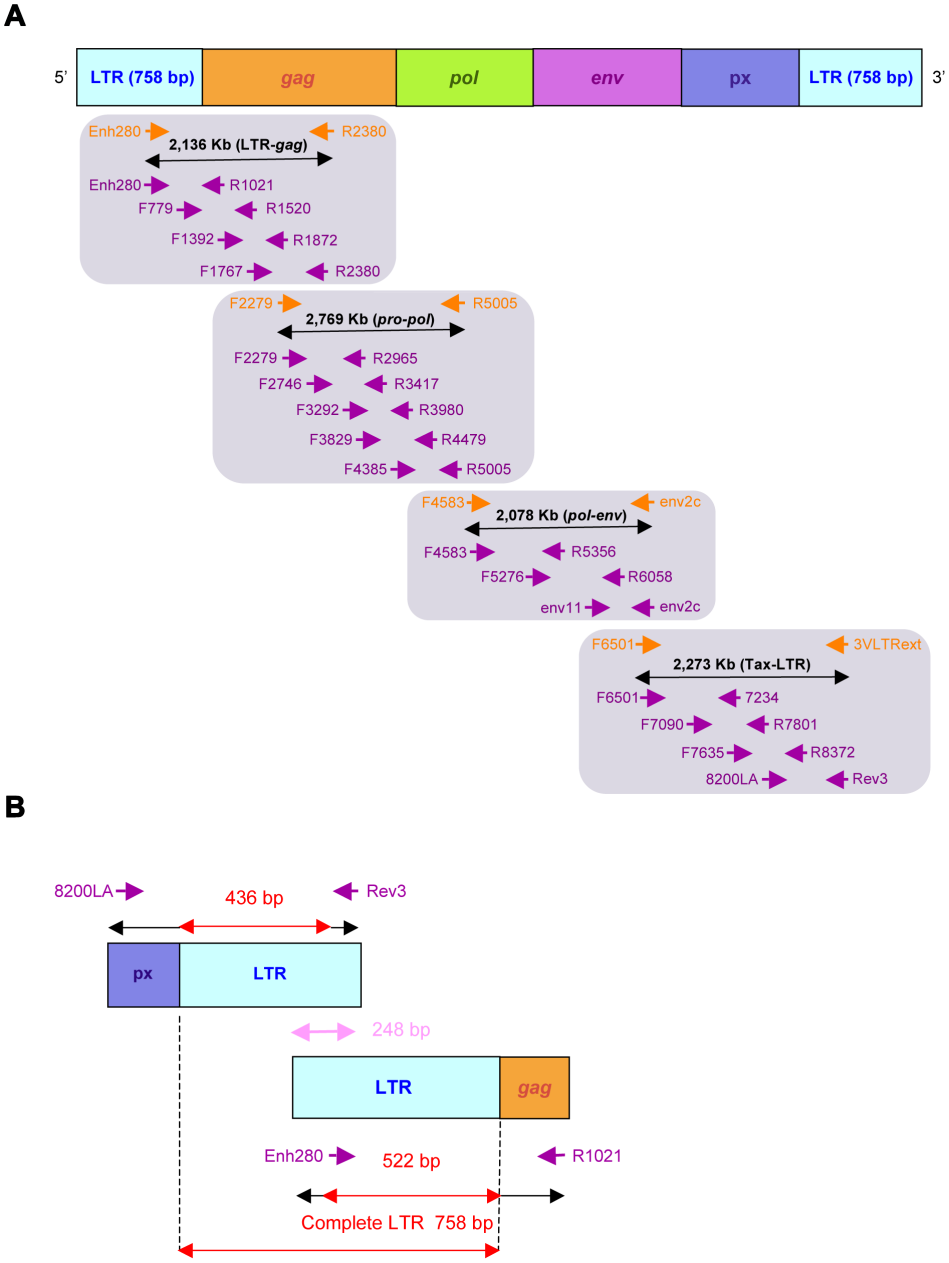
PCR strategy for amplifying both the complete HTLV-1c proviral genome (figure 2A) and the entire Long Terminal Repeat (LTR) region (figure 2B). Both LTR-*gag*, *pro-pol*, *pol-env* and *tax*-LTR fragments, which were amplified by PCR and sequenced are shown (orange arrows) as well as internal primers pairs (purple arrows) designed for sequencing reactions (see table 2 for oligonucleotide primers sequences). Nucleotide numbering is according to the HTLV-1 ATK-1 prototypic sequence (Genbank: J02029).

High-molecular weight DNA was extracted from PBBC using the QIAamp DNA Blood Mini Kit (Qiagen, Hilden, Germany). Samples were first subjected to PCR using human β-globin specific primers, to ensure that DNA was amplifiable [26]. All 27 samples were then submitted to two series of PCR using LTR-*gag* and Px-LTR primers which were designed using highly conserved regions that are common to the major HTLV-1 subtypes (figures 2A and 2B). The LTR-*gag* primers are the following: Enh280: 5′-TGACGACAACCCCTCACCTCAA-3′ and R2380: 5′-GTCCGGAAAGGGAGGCGTATTAG-3′ corresponding to nucleotides 258 to 279 and 2,377 to 2,399 respectively of the prototype ATK-1 sequence (Genbank: J02029). The Px-LTR primers are F6501: 5′-CTTAACTGGGACCTTGGCCTCTCAC-3′ (nt 6,476 to 6,500) and 3VLTRext: 5′-CGCAGTTCAGGAGGCACCRMA-3′ (nt 8,741 to 8,761) (figure 2A).

To obtain the 522-bp fragment of the gp21 *env* gene, 1 µg of DNA from the Aus-GN strain was subjected to 2 series of PCR as previously described [27].

Two additional series of PCR using *pro*-*pol* and *pol*-*env* primers were necessary to obtain the entire genome amplification of the four Australian proviral sequences (Aus-CS, Aus-NR, Aus-DF and Aus-GM). The *pro*-*pol* primers are F2279: 5′-GGAGCAGACATGACAGTCCTTCC-3′ (nt 2,254 to 2,276) and R5005: 5′-GGCGGCTATTAAGACCAGGAAGC-3′ (nt 5,002 to 5,024). The *pol*-*env* primers are the following F4583: 5′-CAGGAGCCATCTCAGCTACCC-3′ (nt 4,560 to 4,580) and *env*2c: 5′-TTTATAAGAGAGTAATGGGGGTATCTG-3′ (nt 6,613 to 6,639) (figure 2A). The size of the different generated amplicons are the following: LTR-*gag*, 2,136-bp; *pro-pol*, 2,769-bp; *pol*-*env*, 2,078-bp and Px-LTR, 2,273-bp (figure 2A).

A PIKO thermocycler (Ozyme, Saint Quentin-en-Yvelines, France) was used with the following amplification conditions. LTR-*gag*: 98°C, 1 mn; 40×(98°C, 10 s; 69°C, 10 s; 72°C, 1 mn); 72°C, 2 mn and Px-LTR: 98°C, 1 mn; 40×(98°C, 10 s; 72°C, 1 mn); 72°C, 2 mn. For *pro-pol*: 98°C, 1 mn; 40×(98°C, 10 s; 68°C, 10 s; 72°C, 1 mn); 72°C, 2 mn and *pol-env*: 98°C, 1 mn; 40×(98°C, 10 s; 62.5°C, 10 s; 72°C, 1 mn); 72°C, 2 mn. Reaction tubes were prepared in a dedicated room outside the laboratory, with a final volume of 50 µl (DNA matrix, 500 ng; dNTP mix (Roche, Basel, Switzerland), 40 µM; 5×Phire reaction buffer which contains 1.5 mM MgCl2 at final reaction concentration (Ozyme, Saint Quentin-en-Yvelines, France), 5 µl; Phire hot start DNA polymerase (Ozyme, Saint Quentin-en-Yvelines, France), 2 U and 0.5 µM of each oligonucleotide primer (Eurofins MWG, Ebersberg, Germany). Five µl of amplified DNA was size fractionated by 1.5% agarose gel electrophoresis, and the PCR products (45 µl) were sent for purification and sequencing reactions to the MilleGen Company (MilleGen, Labège, France) (table 2).

**Table 2:** Oligonucleotide primers used to sequence complete HTLV-1c proviral strains.

Region amplified	Primer name	Sense Primer sequence 5′ – 3′	Region	Primer name	Anti-Sense Primer sequence 5′ – 3′	Sequence size (bp)
LTR-*gag*	Enh280	TGA CGA CAA CCC CTC ACC TCA A	LTR-*gag*	R1021	GGG TAT CCT TTT GGG AGT AG	742
LTR-*gag*	F779	GTT GGG GGC TCG TCC GGG A	LTR-*gag*	R1520	GTA TTC TCG CCT TAA TCC TTG	742
LTR-*gag*	F1392	CCT CCT GCA GTA CCT TTG CTC	LTR-*gag*	R1872	GCA CCG GAA GCA CGG CTG AT	481
LTR-*gag*	F1767	AAT TAC TAC AGG CCC GAG G	LTR-*gag*	R2380	GTC CGG AAA GGG AGG CGT ATT AG	614
*pro-pol*	F2279	GGA GCA GAC ATG ACA GTC CTT CC	*pro-pol*	R2965	TTT AAA CCC TTG GGG TAG	687
*pro-pol*	F2746	CCT GGC GAT TCA TCC ACG ACC	*pro-pol*	R3417	TAA TGA TTG AAC TTG AGA AGG A	672
*pro-pol*	F3292	TCC CGC TGG GCG CTA CCT GAA C	*pro-pol*	R3980	AAG GGG GAA TGA TCT TTG TGA	689
*pro-pol*	F3829	CTG GAG AAC TTT GGA ACA CT	*pro-pol*	R4479	CGC CTT GCC AGA TGT GGT TAG	651
*pro-pol*	F4385	TGC AAG GGG CAA CCA CAA CTG	*pro-pol*	R5005	GGC GGC TAT TAA GAC CAG GAA GC	621
*pol-env*	F4583	CAG GAG CCA TCT CAG CTA CCC	*pol-env*	R5356	TGA TCT GCT GAA AGG GCC AG	774
*pol-env*	F5276	ATT ACA GCC CCA GCT GCT GTA C	*pol-env*	R6058	CAG GAT GAG GGA GTT ATG ACA	783
*pol-env*	Env11	TGG CAC GTC CTR* TAC TCT CCC AAC	*pol-env*	Env2c	TTT ATA AGA GAG TAA TGG GGG TAT CTG	741
*tax-LTR*	F6501	CTT AAC TGG GAC CTT GGC CTC TCA C	*tax-LTR*	R7234	CGG AGG ACC TGC TGG TGG AGG A	734
*tax-LTR*	F7090	TCA CGA TGC GTT TCC CCG CGA G	*tax-LTR*	R7801	GCA GGA GGG GCC AGG TGA TG	712
*tax-LTR*	F7635	CAA CAT TCC ACC CTC CTT CCT CCA	*tax-LTR*	R8372	CTG GGC CCT GAC CTT TTC AGA C	738
*tax-LTR*	8200LA	CTC ACA CGG CCT CAT ACA GTA CTC	*tax-LTR*	Rev3	GGA GGC ACC ACA GGC GGG AGG CG	559

* The corresponding code for the degenerated nucleotide is the following; R = A and G.

### Phylogenetic analyses

Both strands of each PCR product were sequenced, and the ClustalW algorithm (MacVector 6.5 software, Oxford Molecular) was implemented to align forward and reverse sequences of each segment to derive a consensus sequence of the full LTR (758-bp) region, a fragment of the gp21 *env* gene (522-bp), colinearized *gag-tax* (2,346-bp) and *gag-pol-env-tax* (7567-bp) genes (figure 2). Phylogenetic trees were generated from multiple alignments of the LTR region and gp21 *env* together with the colinearized *gag-tax* and *gag-pol-env-tax* genes. Included in the phylogenetic analyses were the 23 new proviral sequences from Australia and the four novel sequences from Vanuatu (ESH18, ESW44, EM5, PE376) that were characterized in the present study together with appropriate sequences of previously characterized strains from PNG (MEL1, MEL2 and MEL7), the Solomon Islands (MEL3 to MEL6 and MEL 8 to MEL10), Vanuatu (PE376, VAN54, VAN136, VAN251, VAN335) and Australia (MSHR-1). Additional representative sequences of the HTLV-1 a, b, d-g subtypes available in Genbank were also included.

The sequences were aligned using the DAMBE program (version 4.2.13) [28]. Absence of saturation of the alignment was confirmed by 2 methods: likelihood mapping (model TN93; non uniform substitution) with Tree-Puzzle software (version 5.2) and the test of Xia and Xie [28] with the DAMBE program. The final alignment was submitted to the Modeltest program (version 3.6) and the best model was selected according to the Akaike information criterion. This was then applied to phylogenetic analyses using the PAUP program (version 4.0b10) to infer trees according to both Neighbor-Joining (NJ) and Maximum Likelihood (ML) methods. To test the robustness of the tree topologies, 1,000 bootstrap replicates were performed. Numbers applied to the nodes of the tree (bootstrap values) indicate frequencies of occurrence for 100 trees. The quartet puzzling algorithm included in the Tree-Puzzle software was applied for the maximum likelihood method [29].

### Divergence time estimation

In order to estimate the divergence time between the different clades, we initially performed a typical molecular clock analysis [9]. This method was not conclusive. Indeed, the sequences seem to be too short (when considering the low mutation rate for HTLV-1) to be informative under the different models. We therefore estimated the divergence time using the previously reported mutation rates for HTLV-1 [30]. A theoretical ancestral sequence was initially determined for each monophyletic clade, and the number of mutation events required to generate the reported current sequences was then calculated. Finally, this average number of mutation events was multiplied by the known HTLV-1 mutation rate [30]. Although the technique is rough, the estimated date for the Vanuatu/Solomon node is 7,440 years BP (31,000–3,700), which is consistent with the date previously proposed (i.e. 10,000 years ago) [9].

## Results

The 23 HTLV-1 infected individuals from Australia included ten women (mean age 49.7 years, range 27–70) and 13 men (mean age 42.2 years, range 16–67) (table 1). The four samples from Vanuatu were obtained from 3 women (mean age 59, range 40–76) and a 61-year old man. All plasma samples exhibited a complete HTLV-1 pattern in WB and the presence of HTLV-1 provirus was investigated in the DNA of these 27 individuals.

### Gp21 env gene fragment analyses

**Figure 3:**
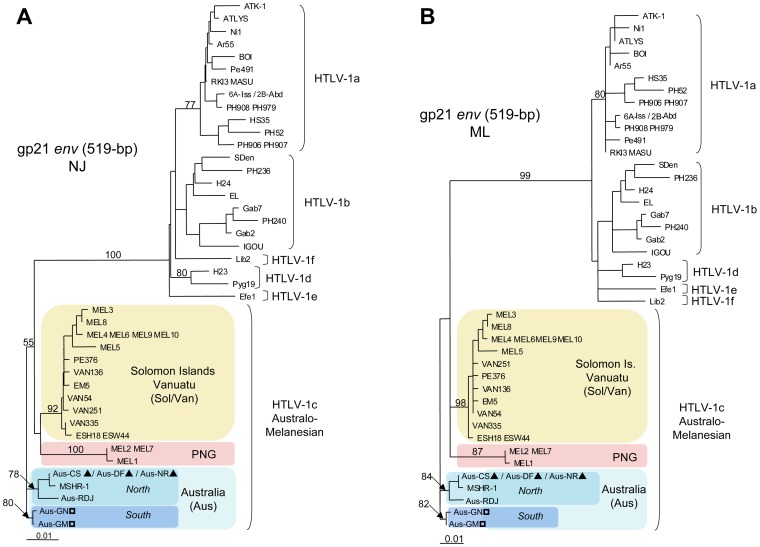
Phylogenetic trees generated with both Neighbor-Joining (NJ) (figure 3A) and Maximum Likelihood (ML) (figure 3B) methods performed in the PAUP program, on a 522-bp fragment of the gp21 *env* gene, for 53 HTLV-1 available sequences including the 5 proviral sequences generated in this work and two previously characterized Australian proviral strains (MSHR-1 and Aus-RDJ). The selected model was the Tamura Nei. a-f correspond to the major HTLV-1 subtypes. HTLV-1c subtype corresponds to the Australo-Melanesian one including the new Australian proviral sequences: Aus-CS, Aus-DF, Aus-NR, Aus-GN and Aus-GM (Genbank: KF242506, KF242505, JX891479, KF242507 and JX891478 respectively) and 2 previously described Australian sequences: MSHR-1 (M92818) and Aus-RDJ (JX891480); 9 sequences from Vanuatu: VAN54, VAN136, VAN251, VAN335, PE376, EM5, PE376, ESH18 and ESW44 (Genbank: AY549880, AY549882, AY549879, AY549881, EF061856, EF061884, EF061856, EF061885 and EF061886 respectively); 7 sequences from the Solomon Islands: MEL3, MEL4, MEL5, MEL6, MEL8, MEL9, MEL10 (Genbank: M94198, M94199, M94200, M93099, U11578, U11580, U11566 respectively) and 3 sequences from Papua New Guinea: MEL1, MEL2 and MEL7 (Genbank: L02533, M94197 and U11576, respectively).

The primary purpose of our work was to study the molecular relationship between the new Australian HTLV-1 proviral strains and those from HTLV-1 infected individuals in Australia and the neighboring islands whose sequences have been previously published. For most strains, the only available sequences in the Genbank database are the 522-bp fragment of the gp21 *env* gene. We therefore compared the gp21 *env* gene fragments of seven proviral sequences from Australia, including five new proviral strains (Aus-Cs, Aus-DF, Aus-NR, Aus-GN and Aus-GM) and two previously characterized sequences (MSHR-1 and Aus-RDJ) (Genbank: M92818 and JX891480, respectively) [15], [25], with HTLV-1 proviral strains from PNG (MEL1, MEL2 and MEL7), Vanuatu (EM5, VAN54, VAN136, VAN251 and PE376) and the Solomon Islands (MEL3 to MEL6 and MEL8 to MEL10) [9], [13], [18], [31]. Phylogenetic analyses performed with both NJ and ML methods clearly demonstrate the existence of three subgroups: “Papua New Guinean”, “Solomon/Vanuatu” and “Australian”, within the HTLV-1c subtype. Furthermore, inside the Australian subgroup, which comprises all the 7 Australian proviral strains, two distinct clades are now observed. The first clade includes strains derived from 3 patients (Aus-CS, Aus-DF and Aus-NR) whose origins are in the North of central Australia plus the two published sequences (MSHR-1 and Aus-RDJ), and the second comprises 2 patients (Aus-GN and Aus-GM) from the South of central Australia (figures 3A and 3B).

### Long Terminal Repeat region analyses

Complete LTR proviral sequences were obtained for all 27 samples by PCR amplification of both LTR-*gag* and Px-LTR fragments. Alignment of the 746-bp LTR fragments for these 27 strains revealed no significant deletion or insertion in comparison to the HTLV-1 ATK-1 reference strain. Within group comparisons of the 23 new Australian HTLV-1 strains indicate that they are closely related to each other (range of nucleotide similarity, 99.5%–100%), though quite divergent from the LTR strains from Vanuatu (nucleotide similarity range, 94.5% – 95.3%) and from the known HTLV-1c subtype prototype strain from the Solomon Islands (MEL5) (nucleotide similarity range, 95.3%–95.5%).

**Figure 4:**
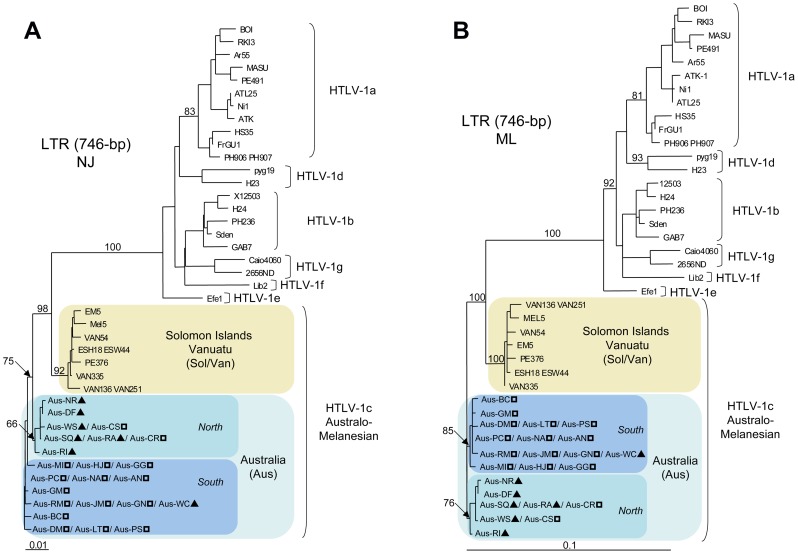
Phylogenetic trees generated with both Neighbor-Joining (figure 4A) and Maximum Likelihood (figure 4B) methods, performed in the PAUP program, on a 746-bp fragment of the LTR region, for 55 HTLV-1 available sequences including the 27 proviral sequences generated in this work. The selected model was the General Time-Reversible (GTR). a-g correspond to the major HTLV-1 subtypes. HTLV-1c subtype corresponds to the Australo-Melanesian one including the 23 new Australian proviral sequences (Genbank: KC786899-KC786919 and JX891478-JX891479) and 4 sequences from Vanuatu (Genbank: KC786962-KC786965). ?, HTLV-1c subtype strains from Northern part of central Australia; ?, HTLV-1c subtype strains from Southern part of central Australia.

Using both NJ and ML methods, the phylogenetic analyses of the LTR region revealed two distinct subgroups within the Australo-Melanesian HTLV-1c subtype. The first group includes strains from the Solomon Islands and Vanuatu (Sol/Van) while the second comprises all the HTLV-1 Australian proviral sequences (Aus) (figures 4A and 4B). Unfortunately, no complete LTR proviral sequence from PNG is available in the sequences databases. However, phylogenetic analysis based on a partial 627-bp fragment of the LTR region, including the PNG-1 strain from Papua New Guinea (Genbank: M85207), clearly confirmed the existence of three distinct clades within the HTLV-1c subtype (data not shown) [32].

Phylogenetic analyses using both gp21 *env* and LTR fragments were consistent with the existence of two Australian HTLV-1c clades. The nature of this relationship was further clarified by comparing larger genomic fragments using both ML and NJ methods.

### Colinearized gag-tax genomic fragment analyses

**Figure 5:**
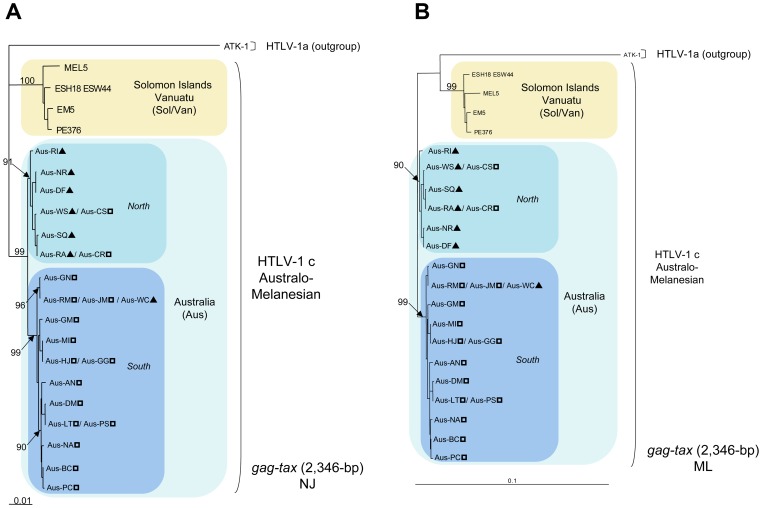
Phylogenetic trees generated with both Neighbor-Joining (figure 5A) and Maximum Likelihood (figure 5B) methods performed in the PAUP program, on a 2,346-bp fragment of the colinearized *gag-tax* genes, for 29 HTLV-1 available sequences including the 27 proviral sequences generated in this work. The selected model was the Tamura Nei. ATK-1 strain was used as an outgroup (Genbank: J02029). The Australo-Melanesian HTLV-1c subtype includes the 23 new Australian sequences characterized in this study (Genbank: KC786920-KC786961 and JX891478-JX891479) and 4 proviral sequences from Vanuatu (Genbank: KC786966-KC786973). ?, HTLV-1c subtype strains from Northern part of central Australia; ?, HTLV-1c subtype strains from Southern part of central Australia.

A comparison of the concatenated and aligned 2,346-bp fragment of the *gag-tax* genes revealed a high degree of nucleotide homology among the Australian strains (range, 98.9%–100%) and a comparable degree of divergence of the Australian strains relative to those from both Vanuatu (range, 94.1%–97.5%) and the Solomon Islands (range, 94.4%–96.9%). Additional phylogenetic analyses using both NJ and ML methods of the colinearized *gag-tax* (2,346-bp) genomic fragment, confirmed the tree topology derived from the gp21 *env* and LTR analyses and demonstrated the existence of an Australian subgroup that was highly supported phylogenetically (bootstrap value ≥ 99%) (figures 5A and 5B). Interestingly, the Australian subgroup can be further subdivided into two clades, for which bootstrap values are also statistically significant (≥ 91%). The first includes HTLV-1 strains derived from most patients of Northern origin (6/7) and the second comprises a majority of individuals (14/16) from the South. Furthermore, a high genetic diversity exists within both Australian clades with sub-clades also supported by high bootstrap values (≥ 90%).

### Colinearized gag-pol-env-tax genomic fragment analyses

These analyses were performed using the complete proviral sequences obtained from four Australian samples: Aus-CS, Aus-NR, Aus-DF and Aus-GM (Genbank: KF242506, JX891479, KF242505 and JX891478 respectively). In addition, two of these complete proviral sequences were selected as the representative prototypes of each Australian clade (“Northern” clade, Aus-NR; “Southern” clade, Aus-GM. The general genomic organization of these two prototypic sequences is similar to that of HTLV-1 prototypes ATK-1 and MEL5 strains (Genbank: J02029 and L02534, respectively). The overall range of nucleotide divergence of the first complete Australian strains from the prototypes ATK-1 and MEL5 was 7.8–8% and 3.3–3.4% respectively. The nucleotide homology between these two Australian prototypic sequences was 98.9% (102 differences over 9,046-bp).

**Figure 6:**
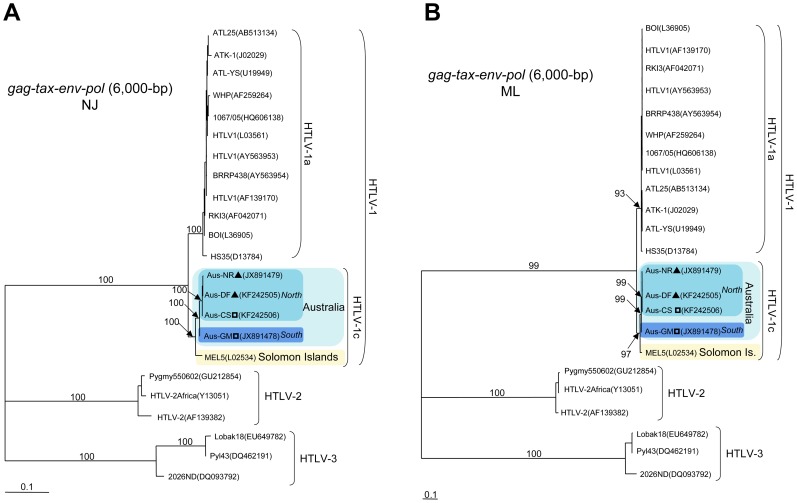
Phylogenetic trees generated with both Neighbor-Joining (figure 6A) and Maximum Likelihood (figure 6B) methods, performed in the PAUP program, on a 6,000-bp fragment of the colinearized *gag-pol-env-tax* genes, for 23 HTLV-1/-2/-3 available sequences including the 4 Australian proviral sequences generated in this work. The selected model was the Tamura Nei. The Australo-Melanesian HTLV-1c subtype includes the 4 complete Australian sequences characterized in this study (Genbank: KC786920-KC786961 and JX891478-JX891479) and the MEL5 proviral sequence from the Solomon Islands (Genbank: L02534). ?, HTLV-1c subtype strains from Northern part of central Australia; ?, HTLV-1c subtype strains from Southern part of central Australia.

Phylogenetic analyses using both NJ and ML methods of the colinearized *gag-pol-env-tax* (6,000-bp) genomic fragments, including HTLV-1, HTLV-2 and HTLV-3 representative sequences available in Genbank, confirmed the existence of an Australian subgroup that was highly supported phylogenetically (figures 6A and 6B).

### Time divergence of the Australian clades

Finally, we estimated the time of divergence for the various Australian strains using the evolution rate of the HTLV-1 LTR region, which has previously been determined by Lemey *et al.* (5.6×10^−7^ substitutions/site/year; 90% confidence interval, 1.2×10^−7^ to 1.1×10^−6^) [30]. These calculations suggest that divergence between the Vanuatu/Solomon and Australian subgroups occurred 20,400 years ago (85,000–10,200) and that speciation of the two Australian clades followed 9,120 years ago (38,000–4,500). The estimated date for the Vanuatu/Solomon node in the present study was 7,440 years ago (31,000– 3,700), which is consistent with our previous estimate (10,000 years ago) [9].

## Discussion

The origin of most HTLV-1 subtypes appears to be linked to ancient and multiple episodes of interspecies transmission between STLV-1-infected non-human primates (NHPs) and humans [5], [33], . Indeed, Old-World NHPs constitute a large reservoir for different lineages of STLV-1, and the virus is considered as transmissible to humans through body fluid contacts [27], [36], [37], [38], [39]. The very high homology between some STLV-1 and HTLV-1 strains, particularly the b and d-f subtypes, suggests that interspecies transmission to humans is probably ongoing in some areas of West and central Africa and results from close contacts during the hunting or butchering of NHPs [8], [37], [38], [40], [41], [42].

Despite the presence of STLV-1-infected NHP species in Asia, there is no evidence of recent interspecies transmission in this area [43]. Furthermore, monkeys have never been endemic to the Australo-Melanesian region, indicating that interspecies transmission of STLV-1 to humans could not have occurred in these islands [44]. Therefore, HTLV-1c is likely to have been acquired by the ancestors of the Indigenous peoples of Australo-Melanesia as a result of interspecies transmission from NHPs during their migration through South-East Asia and prior to reaching the highlands of Papua New Guinea [5], [10], [43], [45]. The subsequent migratory movements of this ancestral population then resulted in the radiation of HTLV-1c throughout the Australo-Melanesian region.

A first wave of migration led to the progressive colonization of the Solomon Islands, followed by the Vanuatu archipelago and finally, New Caledonia and the neighboring Melanesian islands. Consistent with the common origin of these Melanesian populations are our analyses performed on gp21 *env*, the LTR and the colinearized *gag-tax* and *gag-pol-env-tax* genes, which confirm that HTLV-1 strains from the Solomon Islands and Vanuatu belong to the same subgroup. Based on a combination of paleo-anthropological data and genomic DNA analyses, it is believed that the initial human settlement of the Solomon archipelago dates from the Paleolithic period, ca. 30,000 years ago [46], while Vanuatu was settled much latter, during the Neolithic period, ca. 10,000 years ago [47]. In previous phylogenetic and molecular-clock analyses, we suggested that the HTLV-1c proviral strains from the Indigenous people of Vanuatu and the Solomon Islands emerged from a common ancestor ∼10,000 years ago [9], which is consistent with data presented here (ca. 7,440 yrs ago).

A second wave of migration is likely to have occurred from PNG to Australia. Indeed, it is thought that the occupation of Sahul, the continent formed when glacio-eustatically lowered sea levels exposed dry land connections between Australia and Papua New Guinea, may have commenced 45,000 years ago and continued until the end of the Pleistocene period 12,000 years ago [48], [49], [50]. Recently, Rasmussen and colleagues presented evidence derived from the gene flow between populations, which indicates that present-day Indigenous Australians are descendants of the earliest humans to occupy Australia and that they represent one of the oldest continuous populations outside Africa [51].

In the present study, we reveal a high degree of genetic diversity among the HTLV-1c subtype proviral strains that infect the Indigenous people of central Australia. At least two different HTLV-1c genetic clades exist in this Indigenous population and these cluster according to the geographic origin of their human hosts. Thus, it is possible to propose that the common ancestor of the modern Australian HTLV-1 strains arrived in Australia when a group originating from the ancestral HTLV-1 infected population migrated from PNG and settled in Australia. Subsequently, the Australian population split (ca. 9,000 yrs ago) leading to continued viral evolution among small, isolated clan groups of Indigenous people dwelling in the remote desert regions of central Australia and this resulted in the speciation of the two Australian clades. The broad ethno-geographic distinction between the Indigenous human hosts of these Australian clades is particularly interesting given that considerable movement of Indigenous people has resulted from a century of European dominance in this region. Thus, Northern Ngarrkic speaking clan groups were moved to the South while Western Desert groups moved to the East, in each case toward ration supply centers that were established nearer the major regional center of Alice Springs [52]. Prior to colonization contact between these groups is likely to have been minimal. The data presented here therefore describes probably the molecular epidemiological expression of both long-term evolution and more recent human movements that were driven by colonization.

Further studies, which characterize the HTLV-1c proviral strains that infect other Indigenous populations elsewhere in Australia and Oceania, will provide new insights into the origin of these retroviruses, potentially enhancing our understanding of the pathogenicity, evolution and modes of dissemination of these HTLV-1c variants and their human hosts.
